# Web-Based Nursing Intervention for Self-Management of Pain After Cardiac Surgery: Pilot Randomized Controlled Trial

**DOI:** 10.2196/jmir.2070

**Published:** 2012-12-14

**Authors:** Géraldine Martorella, José Côté, Mélanie Racine, Manon Choinière

**Affiliations:** ^1^Faculty of NursingUniversity of MontrealMontreal, QCCanada; ^2^Centre de rechercheCentre hospitalier de l'Université de MontréalMontreal, QCCanada; ^3^Department of psychologyUniversité du Québec à MontréalMontreal, QCCanada; ^4^Faculty of MedicineDepartment of AnesthesiologyUniversity of MontrealMontreal, QCCanada

**Keywords:** postoperative pain, cardiac surgery, patient education, Internet, pilot study, randomized controlled trial

## Abstract

**Background:**

Most adults undergoing cardiac surgery suffer from moderate to severe pain for up to 6 days after surgery. Individual barriers and attitudes regarding pain and its relief make patients reluctant to report their pain and ask for analgesic medication, which results in inadequate pain management. More innovative educational interventions for postoperative pain relief are needed. We developed a Web-based nursing intervention to influence patient’s involvement in postoperative pain management. The intervention (SOULAGE-TAVIE) includes a preoperative 30-minute Web-based session and 2 brief face-to-face postoperative booster sessions. The Web application generates reflective activities and tailored educational messages according to patients’ beliefs and attitudes. The messages are transmitted through videos of a virtual nurse, animations, stories, and texts.

**Objective:**

The aim of this single-blinded pilot randomized trial was to investigate the preliminary effects of a virtual nursing intervention (SOULAGE-TAVIE) to improve pain relief in patients undergoing cardiac surgery.

**Methods:**

Participants (N = 60) were adults scheduled for their first cardiac surgery. They were randomly assigned to the experimental group using SOULAGE-TAVIE (n = 30) or the control group using usual care, including an educational pamphlet and postoperative follow-up (n = 30). Data were collected through questionnaires at the time of admission and from day 1 to day 7 after surgery with the help of a blinded research assistant. Outcomes were pain intensity, pain interference with daily activities, patients’ pain barriers, tendency to catastrophize in face of pain, and analgesic consumption.

**Results:**

The two groups were comparable at baseline across all demographic measures. Results revealed that patients in the experimental group did not experience less intense pain, but they reported significantly less pain interference when breathing/coughing (*P* = .04). A severe pain interference with breathing/coughing (pain ranked ≥ 7/10) was reported on day 3 after surgery by 15% of the patients in the experimental group (4/27), as compared to 44% (7/16) in the control group. On day 7 after surgery, participants in the experimental group also exhibited fewer pain-related barriers as measured by the Barriers Questionnaire-II (mean 10.6, SD 8.3) than patients in the control group (mean 15.8, SD 7.3, *P* = .02). No difference was found for pain catastrophizing. However, in both groups, means revealed a lower tendency to catastrophize pain before surgery as measured by the Pain Catastrophizing Scale (control group mean 1.04, SD 0.74; experimental group mean 1.10, SD 0.95) and after surgery (control group mean score 1.19, SD 0.94; experimental group mean score 1.08, SD 0.99). Finally, the experimental group consumed more opioid medication (mean 31.2 mg, SD 23.2) than the control group (mean 18.8 mg, SD 15.3, *P*  = .001).

**Conclusions:**

This pilot study provides promising results to support the benefits of this new Web-tailored approach that can increase accessibility to health education and promote pain relief without generating more costs.

**Trial Registration:**

Clinicaltrials.gov NCT01084018; http://www.clinicaltrials.gov/ct2/show/NCT01084018 (Archived by WebCite® at http://www.webcitation.org/6CoTBkIoT)

## Introduction

Acute pain is the most commonly experienced pain [1]. October 2010 to October 2011 was the Global Year Against Acute Pain in recognition of its prevalence. Like any pain problem, postoperative pain has physiological, psychosocial, and financial consequences [2-4]. Uncontrolled acute pain results in complications and delayed mobilization of patients after surgery, increased length of stay following surgery, and the risk of chronic pain [1]. It has been estimated that most adults undergoing cardiac surgery suffer from intense pain for up to 6 days after surgery [5-8]. Cardiac surgery, a frequent procedure involving sternotomy, is a source of acute pain and also may contribute to persistent postoperative pain in 17% to 56% of patients [6,9,10]. Analgesic medication is the most common method to relieve pain after this type of surgery, although low doses are often administered [11].

Patients’ attitudes regarding pain and its relief often make them reluctant to report their pain and take analgesic medication [11-13], which could explain inadequate levels of analgesia particularly when patient-controlled analgesia (PCA) is the promoted mode of administration. Moreover, most people expect to suffer from severe pain after cardiac surgery [11]. It has been shown that pain cognitions, such as pain catastrophizing, may influence postoperative pain intensity, activity levels, and analgesic consumption [14,15]. Patients who tend to catastrophize pain may also be hypervigilant and avoid movement, which may cause postoperative complications and persistent pain [16].

Current reviews of traditional nursing educative interventions for surgical populations, including cardiac patients, report unclear objectives and mixed effects on pain [17-19]. Clinically relevant results and statistically significant effect sizes of computer-tailored and Web-based interventions have been recognized for health behavior change with diverse populations [20-22]. Hence, interactive health technologies seem to be powerful and promising media for health education [23-24]. Computers and information technologies have been part of our lifestyle for some time and they can facilitate the implementation of interventions influencing pain behaviors. Computer-tailored interventions have not been integrated into acute pain management approaches, although they seem to be a feasible alternative for surgery. There is a clear lack of innovation in the field of pain education because interventions and conclusions have not changed over almost 20 years [18,19,25]. The challenge is to propose an innovative approach.

**Figure 1:**
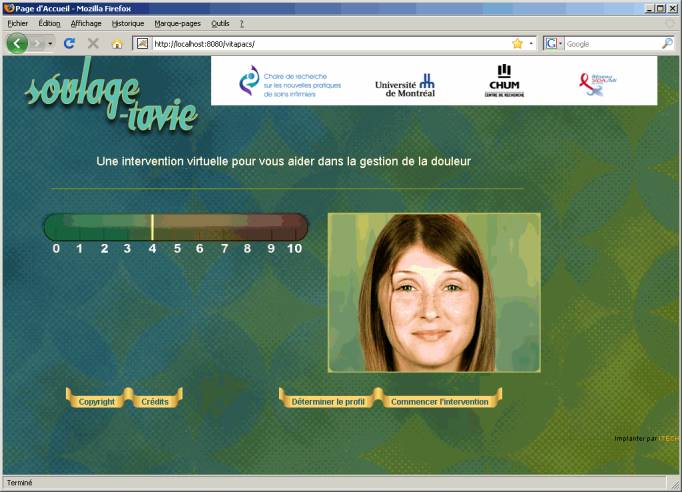
Home page of the SOULAGE-TAVIE website showing the determine profile function and start intervention function.

SOULAGE-TAVIE (*Soutien à l’autogestion - traitement - assistance* *Virtuelle Infirmière - enseignement* or “self-management support - treatment - virtual nursing assistance and education”) was developed by using a pragmatic and evidence-based approach [26]. The Web application was created with the help of a prototype developed by the University of Montréal’s Chair for Research into New Practices in Nursing [27]. Computer-tailored technology was used to offer a complementary and personalized tool to empower patients without adding a burden to the clinicians in the busy environment of acute care. Based on tailored communication [28] and persuasive communication [29] strategies for behavioral change, this tool screens the patient’s pain barriers [30] and tendency to catastrophize pain [31]. It then generates a 30-minute tailored preoperative session on a computer animated by a virtual nurse that guides the participant through the learning process about pain management (Figure 1). Two face-to-face booster sessions of 5 to 10 minutes were also provided. Before this study, the content was validated with clinicians and the Web application’s usability was pretested.

The objective of this pilot study was to assess the preliminary effects of SOULAGE-TAVIE on pain intensity, pain interference with daily postoperative activities, patients’ pain barriers, tendency to catastrophize in face of pain, and analgesic consumption.

## Methods

### Study Design and Randomization Procedure

A single-blinded pilot randomized trial was used to assess the preliminary effects of SOULAGE-TAVIE for patients awaiting cardiac surgery, including coronary artery bypass graft (CABG) or/and valve replacement, during the first week following their operation.

Approval of the protocol was obtained from the University of Montréal Research Committee and from the Ethics Board of the Centre hospitalier de l’Université de Montréal (CHUM). The principal investigator (GM) was responsible of the recruitment and informed consent procedures at the time of admission (usually the day before surgery) and explained the main objective of the study (ie, assessing a new way of educating patients about pain and pain relief) and the components and timing of interventions and follow-up for each group. The randomized allocation through the use of concealed envelopes was also clarified. Each potential participant was given a copy of the informed consent and time to consider whether he or she wanted to participate. After the consent was signed and baseline measures were collected, participants were randomized into 2 groups by the principal investigator: (1) a group to use the SOULAGE-TAVIE application and the usual care procedures (experimental group), and (2) a group to receive solely the usual care procedure, ie, a pamphlet describing general principles of pain management (control group). Permuted-block randomization with an allocation ratio of 4 was used to generate a list through computer software. The list and envelopes were prepared by a colleague who was not involved in this study. An experienced research assistant was blinded and responsible for the face-to-face data collection, except for the medical records that were examined by a trained nurse also blinded to group allocation. Clinical staff was blinded to group allocation and to the roles of the research assistant and principal investigator in the study (data collection vs intervention).

### Participants

Because the pilot study was not expected to be powered to detect statistically significant differences, there is no universal calculation rule to determine sample size. Usually, 20 participants per group is required to be able to make assumptions of homogeneity and normality of variances [32]. However, Hertzog [32] suggests that 30 to 40 patients per group is necessary when no meaningful difference is known and when the researchers would like to proceed to sample size calculation for a larger study. We decided to recruit 60 participants, 30 per group.

Patients were selected according to the following criteria: (1) age 18 years and older, (2) elected for a first-intention cardiac surgery involving sternotomy (CABG, valve replacement, or both procedures) at the cardiac surgery unit of the CHUM, and (3) able to understand and complete questionnaires in French. Patients were not eligible for the study if they (1) had previous cardiac surgery, (2) were planned to be on a postoperative epidural protocol, and/or (3) were unable to consent because of a cognitive or psychiatric disorder.

### Initial Assessment

All participants completed baseline measures in the cardiac surgery unit either a few days before or the day before surgery (T0). Usual sociodemographic variables (ie, age, sex, civil status and living conditions, education level, employment status, and annual income) were collected. Presence of chronic pain before surgery was also documented. Baseline psychological well-being measures were assessed with the Hospital Anxiety and Depression Scale (HADS) [33,34]. The HADS includes 14 items (Likert-type scale ranging from 1 to 4) divided into 2 subscales of anxiety (7 items) and of depression (7 items). Two scores are calculated, but a total score can also be obtained by summing the results of the 2 subscales. The validity and reliability of the HADS is well established [33,35].

### Treatment Conditions

After completing initial measures, all participants received the preoperative education usually provided on the cardiac surgery unit of the CHUM. It consisted of a pamphlet to read in the preoperative phase at the time of admission. This pamphlet presented diverse aspects about the experience of a cardiac surgery. Regarding pain, it explained the use of the pain intensity numeric rating scale (ranging from 0 to 10). It also emphasized the importance of not waiting for the pain to become severe or reaching greater than 4 (out of 10) before asking for analgesic medication or informing the health care staff. Pharmacological and nonpharmacological options were also discussed.

Patients from the experimental group also received the SOULAGE-TAVIE intervention. During the intervention’s development, the elaboration likelihood model [29] guided the choice of 2 strategies to promote attitude change through reflection and deep processing of information. Firstly, messages were built according to tailored communication that included the generation of profiles according to a screening of behavioral determinants, and the combination of different types of feedback (descriptive, comparative or normative, and evaluative) [28]. The messages provided were specifically tailored to the participants’ profile according to real-time answers (dynamic tailoring) as displayed in Table 1, but also according to a predetermined algorithm (static tailoring). The algorithm was based on the mean scores obtained on each of the 7 subscales of the Barriers Questionnaire-II (BQ-II) [30] and the Pain Catastrophizing Scale (PCS) [31], because no cutoff was identified for these tools. However, the use of subscales’ scores instead of total scores allowed the provision of more refined messages. Two profiles (mild vs moderate-high), and consequently 2 types of activities and/or messages, were outlined for each subscale (Table 2). If a score from 0 to 2 was recorded, the application generated a reinforcement message (mild profile). If a score between 2 to 5 on the BQ-II or between 2 to 4 on the PCS was obtained, the application generated a reflection activity (moderate-high profile). Persuasive communication also contributed to the development of messages through the consideration of the source, channel, receiver, and arguments [29]. For example, the source had to be trustworthy and credible. A virtual nurse was chosen as the messenger and different shots were planned depending on the type of messages. Regarding messages, other patients’ experience and research results were used to strengthen arguments on the consequences of behavior and promote self-assessment.

**Table 1:** Example of reflection activity on pain definition based on real-time answers to the question: “What is the pain intensity between 0 and 10 that you expect to feel the day after surgery?”

Feedback	User response	
	0 to 3	7 to 10
Descriptive/comparative	“Between 0 and 3, pain is considered mild. Most people feel moderate to severe pain the first day after cardiac surgery.”	“Some people feel severe pain (between 7 and 10) and, as they expected it, they think it is normal to endure it.”
Reinforcement message through persuasive communication	“You could feel pain higher than 4 if you move for example, although you should target a mild level of pain to facilitate your recovery.”	“Studies recommend maintaining a mild level of pain to promote a good recovery.”
	“Do not let your pain exceed 4!”	“Do not let your pain exceed 4!’’

**Table 2:** Example of tailored message according to score on the Pain Catastrophizing Scale (rumination subscale).

Response	Profile	
	Mild (score ≤ 2)	Moderate-high (score > 2)
Message	When you feel pain, you think about it sometimes but you are able to concentrate on something else. Bravo! It is good to have this attitude when dealing with pain. When people focus their mind on pain, they stop moving, it slows down their recovery, and can also lead to pain elevation.	When you are in pain, you tend to concentrate your mind on it. It is normal because pain is unpleasant! However, by doing so you stop thinking about solutions and you avoid doing your recovery activities to avoid pain.

**Figure 2:**
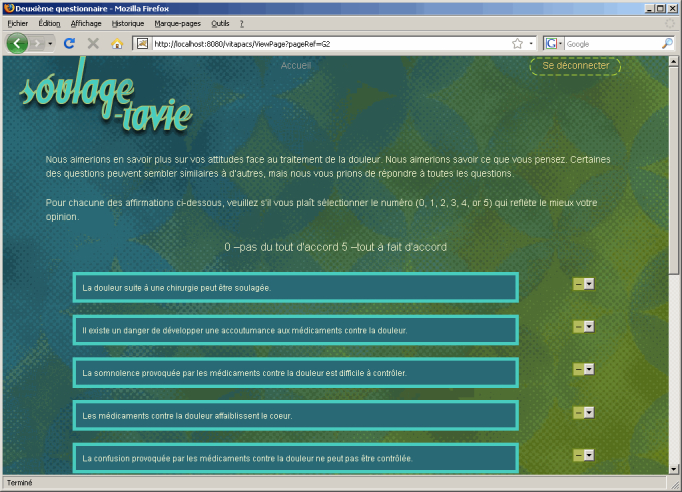
Screening page of the SOULAGE-TAVIE website.

**Figure 3:**
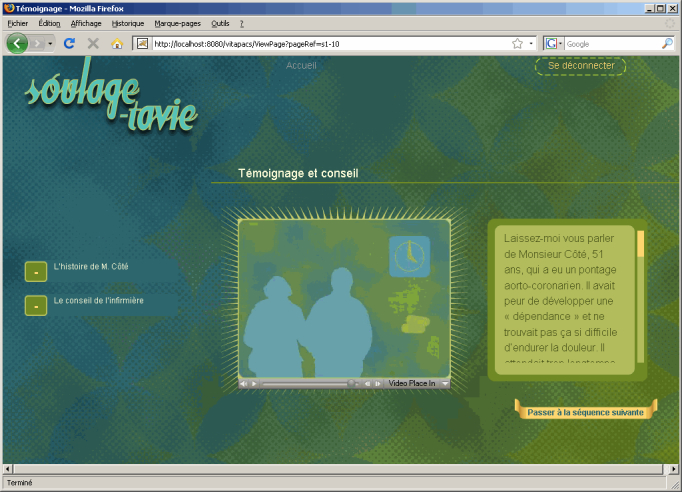
Animated integration page of the SOULAGE-TAVIE website displaying case history and nurse’s advice.

After the screening (Figure 2), the Web session was divided into 3 sequences: definition of pain, individual reaction to pain, and pain management. A total of 47 videos of a virtual nurse were shot and placed on 34 pages, including 4 types of content: 4 screening pages, 15 information pages, 8 question and feedback pages, and 7 integration/consolidation animated pages. The 3 sequences started with an introductory video and general content, followed by activities according to individual scores. Reflective activities included questions and choices of answers. Feedback through a virtual nurse’s advice or an animated video was then provided. Each sequence ended with a video of the virtual nurse or an animation (eg, case history) integrating various elements toward the elaboration of an action plan for postoperative pain (Figure 3). At the end of the session, the virtual nurse reminded the person that he or she would be visited postoperatively by a nurse as a follow-up to the preoperative session.

The Web session usually took place on the surgical unit a few days or the day before surgery through the use of a laptop because a dedicated room and a wireless Internet connection were not available. A nurse (GM) was present to assist participants if technical problems occurred. The 2 boosters were delivered on the surgical unit face-to-face by the principal investigator (GM). The first booster was provided on day 2 after surgery, because this is when patients are usually transferred from the intensive care unit to the surgical unit. The objective was to review the main concepts of medication intake and communication relative to pain level and postoperative activities. The second booster was provided on day 3 after surgery because patients start moving a bit more and the analgesic strategy is usually modified. Moreover, some patients are transferred back to their health centers on day 3. The second booster’s objective was to review specific items based on the preoperative screening of pain barriers and catastrophizing.

### Primary Outcome Measures

Postoperative measures were taken in the intensive care unit (ICU) and in the surgical care unit (SCU).

#### Pain Intensity

Pain intensity was assessed at 24 (day 1), 48 (day 2), and 72 hours (day 3), and at 7 days (day 7) postsurgery using a numerical rating scale (NRS) with a range from 0 to10 (0: no pain at all; 10: worst possible pain) [36,37]. Four different measures of pain intensity were taken: (1) average pain upon movement in the past 24 hours, (2) worst pain upon movement in the past 24 hours, (3) present pain upon movement, and (4) present pain at rest.

#### Pain Interference With Daily Postoperative Activities

As suggested by the Initiative on Methods, Measurement, and Pain Assessment in Clinical Trials (IMMPACT) group in regard to pain core domains in clinical trials [38], the impact of pain on various aspects of daily living was assessed with interference items of the Brief Pain Inventory (BPI) [39,40], which has been successfully validated with cardiac surgery patients [11,41]. It includes 7 items and evaluates the impact of pain on general activity, mood, walking, work, relationships, sleep, and enjoyment of life. Some items were added in the context of the present study to measure the pain-related interference on appetite, concentration, and breathing/coughing. Each item represents a subscale and can be scored and analyzed individually with a range between 0 and 10 (0: does not interfere; 10: completely interferes). A total interference score was also calculated by taking the sum of all the items.

#### Pain Barriers and Catastrophizing

Patients’ barriers toward pain management and tendency to catastrophize were assessed before surgery and intervention (T0) and were reassessed on day 7 after surgery using validated tools. The BQ-II [30] includes 27 items divided into 4 subscales: beliefs regarding secondary effects of medication (12 items), their harmful effects (6 items), fatalism about the control of pain (3 items), and attitudes regarding pain report to health care professionals (6 items). Each item is rated on a 0 to 5 scale (0: totally disagree; 5: totally agree). A total score and scores for each subscale can be calculated by taking the sum of the items. This questionnaire and its subscales have shown internal consistency and sensitivity to change [30,42]. Because a French version of this tool does not exist, we conducted a forward-backward translation protocol [43], and we adapted specific items to the context of cardiac surgery. The final version was reviewed by a group of experts (ie, a psychologist, a physician, and a nurse who were all involved in pain research with the same patients), and tested with 4 patients (2 women and 2 men).

The PCS was used to assess patients’ tendency to catastrophize in the face of pain. It includes 13 items divided into 3 subscales: rumination (4 items), magnification (3 items), and helplessness (6 items). Each item is rated on a 0 to 4 scale (0: not at all; 4 all the time). A total score and scores for each subscale can be calculated by taking the sum of the items. The PCS has demonstrated an excellent internal consistency [31,44] and its sensitivity to psychosocial interventions has been established in the field of chronic pain [45,46].

#### Analgesic Consumption

The analgesic mode of administration (eg, PCA, intravenous injections, and oral medication) was documented. The dose of every opioid received postoperatively was transcribed and converted into standardized parenteral morphine equivalents [47]. A total in milligrams was calculated for each day, and means were obtained and analyzed for both groups at each postoperative day (days 1 to 7).

### Medico-Surgical Assessment

Medico-surgical characteristics (ie, type of surgery and number of grafts, type and length of anesthesia, presence of postoperative complications, duration of ICU stay, and total postoperative length of stay) were assessed to describe sample and compare groups preoperatively and postoperatively.

### Statistical Analyses

The protocol privileged an intention-to-treat approach for the analysis of results. If patients had completed the baseline and one of the postoperative measures, they were included in the study. An alpha = .05 level of significance was used for all analyses. Descriptive statistics (frequency tables, means, and standard deviations) were summarized at each time point. Student’s *t* tests or Chi-square tests were performed for each sociodemographic, medico-surgical, and baseline psychological variables to assure that equivalence of groups was obtained through randomization, although this procedure is not mandatory [48].

The evolution of pain intensity, pain interference, and analgesic consumption in the 7 postoperative days for both groups was examined with 2-way analysis of variance (ANOVA) with repeated measures on 1 factor, such as time with 4 levels (day 1, day 2, day 3, and day 7), and 1 nonrepeated factor (group) with 2 levels (experimental group and control group). The same type of analysis was used to assess the evolution of the patients’ pain barriers and tendency to catastrophize at baseline and on day 7. If interactions were found (*P *< .05), post-hoc comparisons were performed. Independent *t* tests were conducted at each time to compare groups and 1-way repeated-measure ANOVA for each group to study time effects. Chi-square tests were conducted to compare the proportion of patients with pain intensity and pain-related interference ≥ 7/10.

## Results

**Figure 4:**
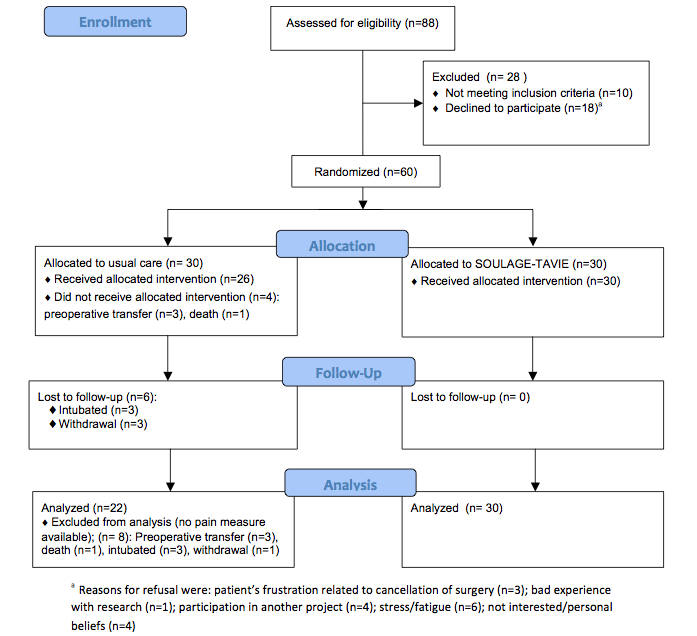
CONSORT flow diagram of participants.

A total of 88 potential participants were approached. Of these, 10 (11%) did not meet the selection criteria (4 were not French-speaking, 1 was deaf, 3 were scheduled for a second surgery, 1 had a stent, and 1 had a cognitive disorder), and 18 (20%) refused to participate (males: 13/18, 72%; females: 5/18, 28%). A sample of 60 patients was recruited over 4 months from February to June 2010. The number of participants at each phase of the trial is illustrated by the Consolidated Standards of Reporting Trials (CONSORT) diagram (Figure 4) [48]. Four patients in the control group did not receive allocated intervention. All patients from the experimental group received SOULAGE-TAVIE (Web session plus 2 booster sessions). Six patients were lost at follow-up in the control group. A total of 52 patients were included in the analysis. Eight patients were excluded from analysis because pain measures were not available.

### Sample Characteristics

Descriptive data for sociodemographic, psychological, and medico-surgical variables are presented in Table 3. The sample included 21% (11/52) of women and 79% (41/52) of men with a mean age of 64 years (range 41-85). No statistically significant differences between the control group and the experimental group were found at baseline for their sociodemographic characteristics. The two groups were comparable in their anxiety level (HADS) before surgery (control group mean 8.32, SD 5.17; experimental group mean 6.77, SD 4.44) and on day 7 after surgery (control group mean 5.84, SD 3.25; experimental group mean 5.37, SD 4.15). The same was true for their depression levels (HADS) prior to surgery (control group mean 2.86, SD 2.29; experimental group mean 3.67, SD 3.40) and after surgery (control group mean 4.21, SD 4.01; experimental group mean 4.30, SD 3.32). No statistically significant differences were found in the medico-surgical variables, except for the number of grafts. The experimental group had a higher number of grafts than the control group. The control group spent more time in the ICU and SCU. These results are explained by one outlier in the control group for both measures. Medians were similar with 17 hours in intensive care for the control group and 14 hours for the experimental group and 7.5 days in postoperative care for the control group and 7 days for the experimental group.

**Table 3:** Demographic and clinical characteristics of the participants in the control and experimental groups.

Variables		Control group n = 22	Experimental group n = 30	*P* value
**Sex, n (%)**				.81
	Female	5 (23)	6 (20)	
	Male	17 (77)	24 (80)	
Age, mean (SD)		63.2 (9.9)	64.6 (8.2)	.58
**Marital status, n (%)**				.89
	Single	1 (4)	1 (3)	
	Married or free union	14 (64)	21 (70)	
	Separated/divorced/widowed	7 (32)	8 (27)	
**Living arrangements, n (%)**				.11
	Lives with spouse (with or without children)	14 (64)	22 (73)	
	Lives with family member or friend	3 (14)	0 (0)	
	Lives alone	5 (22)	8 (27)	
**Education level, n (%)**				.19
	Primary	6 (27)	6 (20)	
	Secondary	6 (27)	8 (27)	
	High school	2 (9)	10 (33)	
	University	8 (37)	6 (20)	
**Employment status, n (%)**				.10
	Full time/part time	10 (45)	14 (47)	
	Unemployed/student	3 (14)	4 (13)	
	Retired	9 (41)	12 (40)	
**Annual income, n (%)**				.77
	< CAD $25,000	9 (41)	10 (36)	
	< CAD $55,000	10 (45)	12 (43)	
	≥ CAD $55,000	3 (14)	6 (21)	
**Presence of chronic pain, n (%)**		9 (41)	10 (33)	.57
Duration of chronic pain in months, mean (SD)		111.3 (157.1)	142.4 (187.5)	.70
**Type of surgery, n (%)**				.48
	CABG	11 (50)	20 (69)	
	Valve replacement (VR)	5 (23)	4 (14)	
	CABG + VR	6 (27)	5 (17)	
Presence of postoperative complication(s), n (%)		15 (68)	13 (45)	.10
Use of patient-controlled analgesia, n (%)		13 (59)	17 (59)	.97
Number of grafts, mean (SD)		2.47 (1.0)	3.29 (1.1)	.02
Anesthesia duration in minutes, mean (SD)		204.9 (82.6)	210 (70.2)	.82
Opioid dose during surgery expressed into morphine equivalents, mean (SD)		51.0 (36.7)	50.3 (35.7)	.95
Intensive care length of stay in hours, mean (SD)		84.7 (202.7)	32.0 (24.8)	.17
Postoperative length of stay in days, mean (SD)		11.2 (9.6)	7.5 (3.3)	.06

### Pain Intensity

Statistical analyses revealed no significant group by time interactions for the 4 pain intensity measures (average and worst pain upon movement in the past 24 hours, present pain upon movement, and present pain at rest). Pain intensity scores decreased significantly over time in both groups (*P* = .001). Because the experimental group had significantly more grafts than the control group, a repeated-measures analysis of covariance (ANCOVA) was performed including the number of grafts as a covariate for pain intensity results. Conclusions were similar to those of the ANOVA. No statistically significant difference was found between groups in the proportion of participants suffering from severe pain (intensity ≥ 7/10) on the 4 measures of pain for each time point.

### Pain Interference With Daily Postoperative Activities

No significant group by time interactions were found for the total pain interference BPI scores. The same was true for each subscale of the BPI measuring pain interference with different aspects of daily living. The items *walking* and *appetite* were removed from analysis at day 1; most patients did not answer this item because it did not apply to their condition. However, patients of the experimental group tended to report that their pain interfered less with deep breathing and coughing (*F*_1,31_ = 4.09; *P* = .05), as expressed by their postoperative mean on this subscale at each time (day 1 mean 4.7, SD 2.5; day 2 mean 4.9, SD 2.7; day 3 mean 3.6, SD 1.9; and day 7 mean 3.4, SD 2.6) compared to the control group (day 1 mean 6.2, SD 2.8; day 2 mean 6.1, SD 3.2; day 3 mean 5.4, SD 3.8; and day 7 mean 5.0, SD 3.8).

A second set of analyses was carried out to compare the percentage of patients in each group who reported severe pain interference (score ≥ 7/10) on the different subscales of the BPI. As shown in Table 4, a statistically significant difference in favor of the experimental group was found on the deep breathing and coughing subscale on day 3 (*P* = .04) and a result close to statistical significance emerged on day 7 (*P* = .06). A significantly lower percentage of patients in the experimental group also reported severe pain-related interference on their appetite on day 7 when compared to the control group (*P* = .02). Results close to statistical significance were also observed in the experimental group with regards to pain interference with walking (*P* = .06) and concentration (*P* = .06) on day 2 postoperatively.

**Table 4:** Number and percentage of patients who reported severe pain interference (≥ 7/10) in specific activities as assessed by the Brief Pain Inventory (BPI) in the control and experimental groups.

Activities		Day 1		Day 2		Day 3		Day 7	
		n/N (%)	*P* value	n/N (%)	*P* value	n/N (%)	*P* value	n/N (%)	*P* value
**Walking**			n/a^a^		.06		.19		.42
	Experimental group	n/a^a^		2/21 (9)		3/27 (11)		4/30 (13)	
	Control group	n/a^a^		5/14 (36)		4/15 (27)		4/18 (22)	
**Appetite**			n/a^a^		.50		.98		.02
	Experimental group	n/a^a^		4/26 (15)		5/27 (18)		1/30 (3)	
	Control group	n/a^a^		4/17 (23)		3/16 (19)		5/19 (26)	
**Concentration**			.18		.06		.23		.93
	Experimental group	4/25 (16)		3/26 (11)		3/27 (11)		5/30 (17)	
	Control group	6/18 (33)		6/17 (35)		4/16 (25)		3/19 (16)	
**Breathing and coughing**			.23		.28		.04		.06
	Experimental group	8/25 (32)		8/26 (31)		4/27 (15)		3/30 (10)	
	Control group	9/18 (50)		8/17 (47)		7/16 (44)		6/19 (31)	

^a^ n/a: not applicable to the patients’ condition

### Pain Barriers and Catastrophizing

A significant group by time interaction was found for attitudes related to harmful effects of analgesic medication (*F*_1,46_ = 5.61; *P* = .02), as shown in Table 5. Post-hoc tests revealed that the experimental group had significantly fewer of these barriers than the control group at day 7 (*P* = .03). Since groups were not significantly different at baseline (*P* = .61), it seems that they experienced a different evolution after surgery that made them significantly different at day 7.

**Table 5:** Mean scores on the Barriers Questionnaire-II (BQ-II) for the control and experimental groups.

		Baseline, mean (SD)		Day 7, mean (SD)		*P* value		
		Experimental	Control	Experimental	Control	Group	Time	Interaction
**Subscales of BQ-II**								
	Secondary effects	20.4 (12.4)	25.1 (12.2)	17.7 (14.7)	26.9 (15.1)	.06	.81	.22
	Harmful effects	12.6 (8.0)	13.4 (8.5)	10.6 (8.3)	15.8 (7.3)	.18	.80	.02
	Fatalism	1.8 (2.5)	1.7 (2.6)	2.2 (2.5)	0.8 (1.0)	.23	.46	.07
	Communication	11.1 (8.5)	8.2 (6.4)	9.7 (7.6)	10.6 (7.7)	.62	.65	.07
Global score on the BQ-II		45.9 (25.9)	47.9 (19.5)	40.2 (29.4)	53.3 (27.7)	.29	.95	.07

The experimental group exhibited fewer pain-related attitudes on day 7 than the control group, although this did not meet statistical significance (*P* = .07). Since groups were equivalent at baseline (*P* = .36), patients of the experimental group tended to exhibit fewer pain-related attitudes at day 7, as expressed by their means (prior to surgery mean 45.9, SD 25.9; day 7 mean 40.2, SD 29.4) compared to the control group (prior to surgery mean 47.9, SD 19.5; day 7 mean 53.3, SD 27.7). Since the study involved a restricted sample, this result may suggest a lack of power to detect a treatment effect on global pain-related barriers. A power calculation was then run regarding the evolution of means for the global score on the BQ-II between day 2 and day 7 after surgery, group sample sizes of 56 (N = 112) achieve 80% power to detect a difference in mean scores with a significance level (alpha) of .05 by using a 2-sided 2-sample *t* test.

Results obtained on the PCS revealed no group by time interaction. However, mean scores for both groups suggest that patients showed a low tendency to catastrophize in face of pain before (control group mean 1.04, SD 0.74; experimental group mean 1.10, SD 0.95) and after surgery (control group mean 1.19, SD 0.94; experimental group mean 1.08, SD 0.99).

### Analgesic Consumption

As seen in Table 6, results of the statistical analysis revealed a group by time interaction with regard to opioid consumption after surgery indicating that the intake was higher in the experimental group than the control group (*F*_6,240_ = 4.06; *P* = .001). However, post-hoc tests revealed that the group difference was statistically significant only on day 2 (*P* = .006).

**Table 6:** Opioid dose after surgery expressed into milligrams (mg) of morphine equivalents for both control and experimental groups.

Postsurgery day	Opioid dose (mg morphine)		*P* value
	Control group mean (SD)	Experimental group mean (SD)	
Day 1	21.9 (13.4)	26.4 (16.2)	.65
Day 2	18.8 (15.3)	31.2 (23.2)	.006
Day 3	13.3 (12.6)	17.7 (15.4)	.11
Day 7	3.2 (4.5)	4.3 (7.1)	.57

## Discussion

This study examined the preliminary effects of a Web-based nursing intervention for postoperative pain after cardiac surgery and showed promising results supporting the short-term benefits of SOULAGE-TAVIE for improving important postoperative pain-related outcomes. Our findings showed that patients who received the intervention reported significantly less pain interference when breathing and coughing, exhibited fewer pain-related barriers, and consumed more opioid medication than those of the control group. However, delivery of the intervention did not translate into less-intense postoperative pain.

### Significant Results

Pain severity can be assessed by its intensity and also by its impact on various aspects of daily living [38,49]. In the present study, no group difference was found for pain intensity, but patients of the control group reported significantly more pain interference with breathing/coughing. An earlier randomized controlled trial (RCT) [11] evaluated the effects of an educative pamphlet with the same population. They recorded a difference between groups regarding pain impact on breathing/coughing on day 5. However, in the current study, the difference was observed earlier (day 3) and results suggested that this tendency was maintained until day 7. At the usual time of discharge (day 7), patients from the control group still experienced a moderate level of pain interference with breathing/coughing compared to a mild level for the experimental group. Important results from a clinical point of view were also found for concentration, appetite, and especially walking. Breathing/coughing and walking are practiced early in the postoperative phase and are crucial activities for patients’ recovery [50,51].

Because SOULAGE-TAVIE was meant to promote self-management, one of the most interesting results is that it had an effect on analgesic consumption. Several studies underlined the lack of analgesia in the surgical population [8,11,12]. Until now, no intervention, even when targeting pain-related barriers, had an effect on opioid intake [11,42]. Results of our study revealed that, compared to the control group, the experimental group consumed significantly more opioids on day 2 (ie, after their transfer from intensive care) although modes of analgesia required more involvement (PCA vs as needed). For that matter, a booster session of SOULAGE-TAVIE was given at that time point. A difference of 60% in the opioid consumption was recorded when patients started moving more (day 2).

The presence of pain barriers has already been associated with a low analgesic intake [30,42,52]; therefore, it is not surprising that the intervention also modulated the evolution of attitudes toward harmful effects of medication (ie, one of the targets of the intervention). Indeed, the experimental group exhibited significantly fewer of these pain-related attitudes on day 7. A previous RCT on an educative intervention (pamphlet and group meeting) in the same population had found a significant difference between groups on some negative pain-related attitudes on day 5 [11]. Another RCT tested an individualized intervention targeting pain-related barriers in persons suffering from cancer pain and showed a greater decrease of these attitudes in its experimental group [42]. These studies reported interesting results, but the present one showed that groups evolved differently after their surgery.

It is difficult to delineate the specific contribution of the intervention’s components. However, some principles were considered during the development of SOULAGE-TAVIE and can be taken into account. It should first be noted that the elaboration likelihood model and predictors, such as pain barriers and catastrophizing, were used to select intervention techniques and develop messages. This procedure was found to be more effective to influence behavior change, particularly with Internet-based interventions [22,28]. The main difference between SOULAGE-TAVIE and previous tested interventions (standardized, individualized) for pain relief is the computer-tailoring approach that improves health behaviors through the delivery of highly personalized messages [20,24,28]. Such messages stimulate the motivation to reflect on attitudes and suggested behavior [28,29]. This thoughtful process is associated to higher persistence of attitude change, stronger resistance to counter-persuasion and consistency between attitude and behavior [29,53]. The combination of computer-tailoring and persuasive communication techniques generated the use of various strategies to build messages, which was also found to increase the effect of Internet-based interventions [22]. SOULAGE-TAVIE used three strategies of tailoring (personalization, content matching, and feedback). The combination of these strategies increases the consideration of messages [20,28,54]. However, feedback seems to be the most efficacious [20,28]. The combination of various types of feedback, ie, descriptive, comparative (normative), and evaluative, is also known to be more beneficial [20,24,28]. Moreover, promoting social comparison (comparative feedback) and providing feedback on performance (evaluative feedback) through Internet-based interventions was found to influence behaviors [22].

Interactive health technologies (IHT) also contributed to the success of SOULAGE-TAVIE because of their attractiveness, diversity, and flexibility [23,27,55]. The SOULAGE-TAVIE application allowed the mix of modalities (animation, quiz, case history, and virtual nurse’s advice) that helped to avoid redundancy of messages and to keep the participant’s attention [23,24,29]. The virtual nurse was an original way to convey educational messages, because computer-tailored messages are still primarily transmitted in a written format even when the intervention is provided through the Internet [20,24]. The goal was not to replace a real patient-nurse relationship, but the personification of feedback was meant to give the sense of an interaction and personalized consultation [27]. Personal contact seems to support behavior change in Internet-based interventions [22]. Finally, because of IHT, not only static tailoring (predetermined algorithm) was possible, but dynamic tailoring (in real time) was also provided, which has already been associated with larger effects on behaviors [20].

### Nonsignificant Results and Limitations

As mentioned, pain intensities were not affected by our intervention as observed earlier [11]. Some authors highlighted unspecific effects in intervention research that could explain this phenomenon, such as therapeutic alliances but also patients’ expectations [56-58]. However, this result is more surprising in this study, because the experimental group consumed more opioid medication. This finding could be explained by the fact that the experimental group experienced less pain interference and consequently practiced more postoperative activities resulting in more pain. Hence, from a clinical point of view, the experimental group reported less postoperative complications than the control group (45% vs 68%).

The tendency to catastrophize in face of pain was found to be quite low in both groups of patients and the recruitment timing might explain this phenomenon. Patients were recruited at the time of admission on the cardiac surgical unit. The announcement of their diagnosis and open-heart surgery was often made a few hours before as the surgeons avoided the use of a waiting list. Pain catastrophizing has been studied in elective cardiac surgical patients, but authors did not underline the timing of the announcement versus recruitment [15]. Other authors studied pain catastrophizing in relation to postoperative pain with a variety of clienteles excluding emergency and cardiac surgical patients [14] (ie, in patients who are in a less life-threatening situation).

The present study has some limitations. With respect to internal validity, unblinding of the research assistant could have occurred although the data collection took place on two departments (ICU and SCU) and at different times than the intervention. Contamination was possible postoperatively during boosters, but the main content was given preoperatively through the Web session. It should also be noted that the intervention was always provided by the same person. This may have increased uniformity in the intervention’s delivery, but increased the possibility of a practitioner effect on patient outcomes as well [57,59,60].

### Future Research

SOULAGE-TAVIE is a first and promising attempt at educating people on pain relief, particularly in the acute care setting, as recent reviews on computer-tailored and Web-based interventions do not report interventions targeting pain [20,24]. Research avenues are numerous with regard to this approach because of computer tailoring and IHT. For instance, the influence of sociodemographic variables (eg, sex differences) on learning and clinical outcomes has been observed in the cardiac surgery population [61-63]. Because it was not possible to examine these differences in the context of a pilot study with a restricted sample, it would be interesting to further explore these patients’ characteristics in a large-scale study on the efficacy of SOULAGE-TAVIE. The influence of mediators and/or moderators related to delivery of computer-tailored interventions through IHT has not really been investigated [20]. Again, individual characteristics and related preferences could influence the impact of this media. In the case of SOULAGE-TAVIE, there is a need to examine whether the presence of the virtual nurse complemented the benefits of the highly personalized messages.

### Conclusions

In conclusion, it seems possible to influence pain management behavior with a brief intervention if educative messages are tailored and personally relevant for the individual. The findings of this pilot RCT provide promising support for the benefits of a Web-based and tailored nursing intervention on postoperative pain management. In contrast to other educational approaches for pain management, SOULAGE-TAVIE included specific mechanisms and strategies of personalization and feedback. The preliminary effects are encouraging enough to warrant further efficacy and long-term effectiveness evaluation of this new educational tool.

Nursing holds a privileged place to intervene in the primary prevention of pain. Improving health information before and after surgery can decrease barriers to pain management through patient empowerment and self-management of pain [33]. Since this intervention could be offered on the Web, this format can increase accessibility to health education without generating more costs [8,42,51]. The development of new and cost-efficient ways to care for patients with acute pain, the most commonly experienced pain, is crucial to decrease the gap between evidence and practice results of undertreatment [32,35].

## Multimedia Appendix 1

CONSORT-EHEALTH checklist V1.6.1. [64].

## Abbreviations

ANCOVA: analysis of covariance
ANOVA: analysis of variance
BPI: Brief Pain Inventory
BQ-II: Barriers Questionnaire-II
CABG: coronary artery bypass graft
CHUM: Centre hospitalier de l’Université de Montréal
HADS: Hospital Anxiety and Depression Scale
ICU: intensive care unit
IHT: interactive health technologies
IMMPACT: Initiative on Methods, Measurement, and Pain Assessment in Clinical Trials
NRS: numerical rating scale
PCA: patient-controlled analgesia
PCS: Pain Catastrophizing Scale
RCT: randomized controlled trial
SCU: surgical care unit
VR: valve replacement
